# Great tits (*Parus major*) flexibly learn that herbivore‐induced plant volatiles indicate prey location: An experimental evidence with two tree species

**DOI:** 10.1002/ece3.7869

**Published:** 2021-07-21

**Authors:** Katerina Sam, Eliska Kovarova, Inga Freiberga, Henriette Uthe, Alexander Weinhold, Leonardo R. Jorge, Rachakonda Sreekar

**Affiliations:** ^1^ Biology Centre of Czech Academy of Sciences Institute of Entomology Ceske Budejovice Czech Republic; ^2^ Faculty of Sciences University of South Bohemia Ceske Budejovice Czech Republic; ^3^ Molecular Interaction Ecology Institute of Biodiversity Friedrich Schiller University Jena Jena Germany; ^4^ Molecular Interaction Ecology German Centre for Integrative Biodiversity Research (iDiv) Halle‐Jena‐Leipzig Leipzig Germany; ^5^ School of Biological Sciences National University of Singapore Singapore Singapore

**Keywords:** Avian olfaction, foraging, herbivore‐induced plant volatiles, HIPVs, induced indirect plant defense, insect herbivores, insectivorous birds, multitrophic interactions, *Parus major*

## Abstract

1. When searching for food, great tits (*Parus major*) can use herbivore‐induced plant volatiles (HIPVs) as an indicator of arthropod presence. Their ability to detect HIPVs was shown to be learned, and not innate, yet the flexibility and generalization of learning remain unclear.
2. We studied if, and if so how, naïve and trained great tits (*Parus major*) discriminate between herbivore‐induced and noninduced saplings of Scotch elm (*Ulmus glabra*) and cattley guava (*Psidium cattleyanum*). We chemically analyzed the used plants and showed that their HIPVs differed significantly and overlapped only in a few compounds.
3. Birds trained to discriminate between herbivore‐induced and noninduced saplings preferred the herbivore‐induced saplings of the plant species they were trained to. Naïve birds did not show any preferences. Our results indicate that the attraction of great tits to herbivore‐induced plants is not innate, rather it is a skill that can be acquired through learning, one tree species at a time.
4. We demonstrate that the ability to learn to associate HIPVs with food reward is flexible, expressed to both tested plant species, even if the plant species has not coevolved with the bird species (i.e., guava). Our results imply that the birds are not capable of generalizing HIPVs among tree species but suggest that they either learn to detect individual compounds or associate whole bouquets with food rewards.

## Graphical abstract

Naïve birds did not show a significant preference for herbivore‐induced saplings, while elm and guava‐trained birds preferred herbivore‐induced saplings of the species on which they were trained in most of the cases.

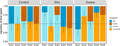

## 1 INTRODUCTION

Plants attacked by herbivorous arthropods release volatiles (herbivore‐induced plant volatiles, HIPVs) that might be used by natural enemies of the herbivores to locate prey or hosts (Dicke, 2015; Dicke et al., 1990, 2009; Vet & Dicke, 1992). The HIPVs that plants release in response to herbivory vary depending on the plant species being attacked as well as the insect herbivore species and its density (Cai et al., 2014; De Moraes et al., 1998; Dicke et al., 1998; Girling et al., 2011; Hare, 2011; Mumm & Dicke, 2010; Pisani Gareau et al., 2013). It is mostly unknown if predators use of olfactory signals while searching for prey is an innate or learned behavior. Some studies have shown that learned olfactory signals are used by insects (Steidle & Van Loon, 2003; Vet & Dicke, 1992), rabbits (Semke et al., 1995), and fish (Nevitt & Dittman, 1998). On the other hand, use of olfaction by parasitoids in the search for their host is an innate trait (Dicke, 2015; Dicke & van Loon, 2000).

Birds have also been shown to be attracted to trees infested by herbivores, without seeing the insects or physical foliage damage (Mäntylä et al., 2004, 2008, 2016, 2020). Yet, only recently we started to disentangle the mechanism underlying the use of olfactory signals by birds searching for prey (Amo et al., 2013; Koski et al., 2015; but see Koski et al., 2015). Despite existing evidence that the ability might be learned in birds, robust experimental work is required to determine whether bird's ability to recognize volatile compounds is a really innate or a learnt trait.

Existing evidence suggests that the use of olfaction in foraging bird species can be an innate or learnt trait depending on the bird species and type of cue. Some bird species, after experience with volatile compounds, can associate such compounds with a particular food source (Caspers et al., 2013; Cunningham & Nevitt, 2011; Gwinner & Berger, 2008; Mennerat et al., 2005; Sneddon et al., 1998). Naïve birds have been shown to not have any preference for the olfactory signal of herbivore‐induced apple saplings compared to the olfactory signal of noninduced saplings (Amo et al., 2016); however, after gaining experience of foraging for caterpillars in trees, they exhibited a preference for the olfactory signal of herbivore‐induced saplings (i.e., caterpillar‐infested trees) showing that learning can occur (Amo, Jansen, et al., 2013). On the other hand, in some procellariform and sphenisciform bird species, the use of olfaction in foraging is an innate trait (Amo et al., 2013; Bonadonna et al., 2006). Innate recognition of more simple chemical cues (e.g., atmospheric dimethyl sulfide for marine birds) might be however more likely than innate recognition of variable and complex HIPVs.

While the innate detection of HIPVs may be under strong selection pressure for specialized species (e.g., parasitoids), generalist predators may need to adapt their foraging behavior in response to changes in the availability, distribution, and abundance of prey species (e.g., Murakami, 1998, 2002). Under these circumstances, natural selection may have favored the ability to flexibly and quickly learn to associate different scents with different food resource in order to maximize foraging (Royama, 1970) or generalize based on typical compounds. The HIPVs are typically complex, yet some compounds are shared between many plant species, but in different proportions (Dicke & Baldwin, 2010). These co‐occurring compounds could serve as a generalized cue revealing presence of prey to generalist predators, if birds are capable of generalization, and do not need to use complex bouquets of HIPVs during the potential learning process.

**FIGURE 1:**
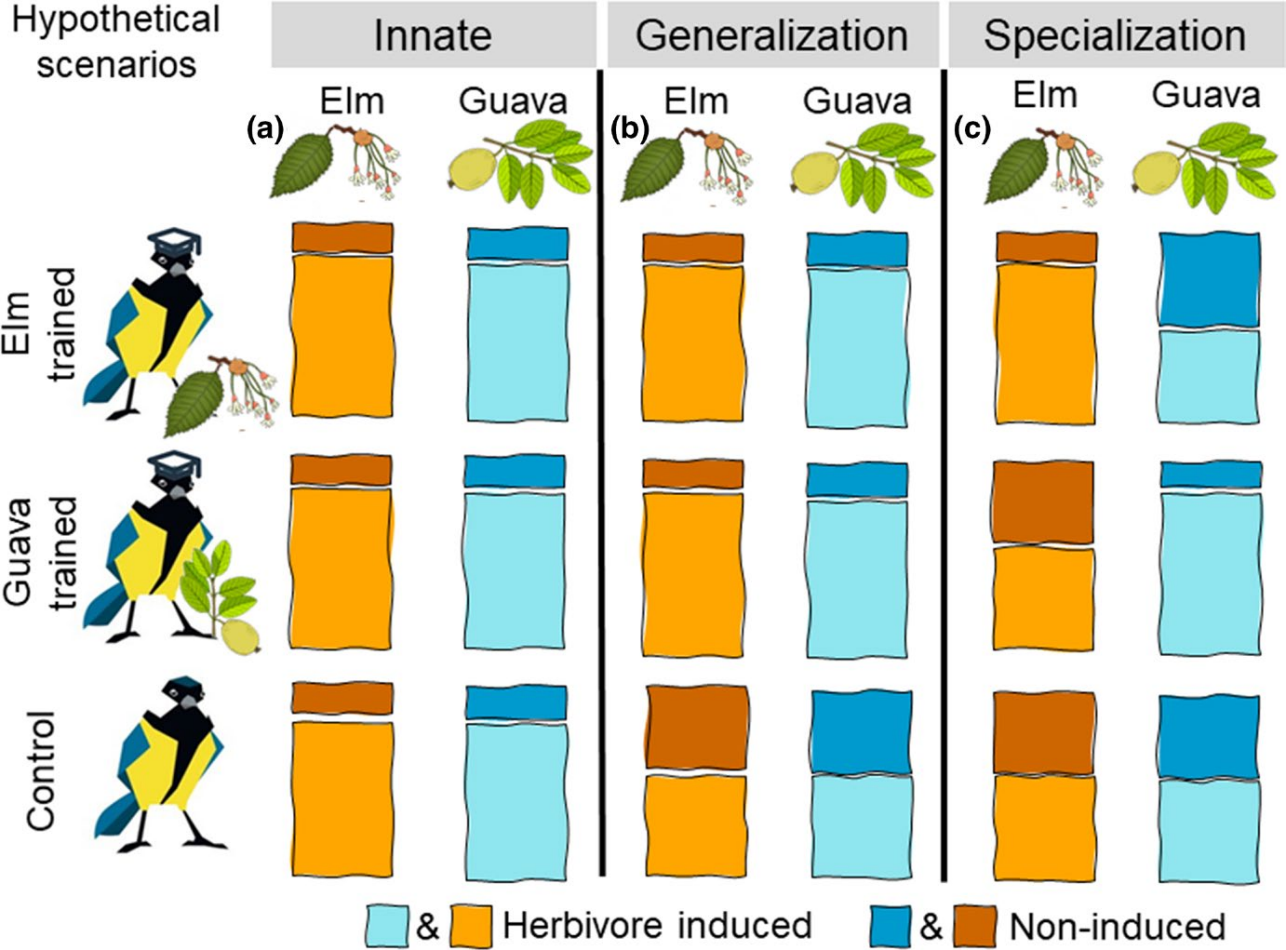
Hypothetical scenarios for results: (a) The ability to distinguish between herbivore‐induced and noninduced plant individuals is innate; thus, both trained and untrained birds spend more time by searching for prey in herbivore‐induced saplings of any plant species. (b) The birds might need learning but are capable of generalization based on the presence of HIPVs shared across plant species. Only trained birds thus prefer herbivore‐induced saplings of any plant species. (c) Birds need to learn specific odors and associate them with reward through learning process. Thus, birds trained to a certain herbivore‐induced plant species will prefer only this familiar specific plant species and only if it is herbivore‐induced. More alternative scenarios are mentioned in the introduction

No previous study has aimed at teaching birds to associate food reward with specific HIPVs bouquets and test the response of both trained and naive birds to the familiar and the novel bouquets of HIPVs. In our experiment, we aimed to investigate whether great tits (*Parus major*) are able to learn to associate HIPVs of specific plant species with food reward, if so, how quickly this can be learnt, and whether they are capable of generalizing the obtained knowledge to different plant species based on the subset of shared HIPVs. We expected one of four possible scenarios. First, the ability to distinguish between herbivore‐induced and noninduced plant individuals might be innate thus both trained and untrained birds would spend more time by searching for prey in herbivore‐induced saplings of any plant species. In this case, we predicted that naïve birds would not show any preference for any saplings, regardless of olfactory signal produced by them (Figure 1a). Second, the birds might need learning but are capable of generalization based on the presence of HIPVs shared across plant species. Under this scenario, only trained birds would prefer to search for food in herbivore‐induced saplings of any plant species offered to them, as long as they share some HIPV compounds based on which the birds can generalize (Figure 1b). Third, birds need to learn specific odors and associate them with reward through learning process. Thus, birds trained to a certain herbivore‐induced plant species will prefer only this familiar specific plant species and only if it is herbivore‐induced (Figure 1c). Alternative to the second scenario, it could be that birds are capable of generalization but based on different HIPVs than the studied here. In that case, we would observe results similar to scenario three (as in Figure 1c), but our conclusions would be false. Fourth scenario might be that the ability to distinguish between herbivore‐induced and noninduced plant individuals might be innate but only to the evolutionary familiar plant species. While this scenario is plausible, it is outside of the scope of the current study, and our design does not allow to study it.

**FIGURE 2:**
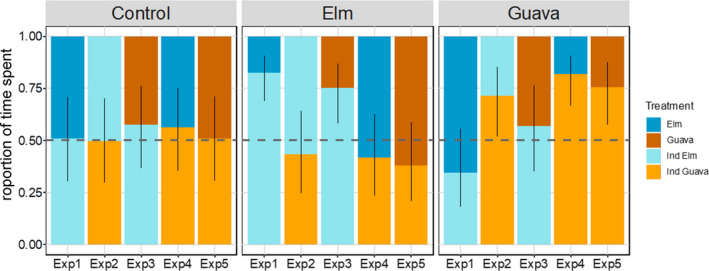
The preference of birds toward individual treatments, estimated by a generalized linear mixed model. The proportion of time spent by birds with different training on each sapling across all experiments. Naïve (control) birds did not show a significant preference of either sapling, while elm and guava trained birds preferred herbivore‐induced saplings of the species on which they were trained, except for Exp 2 for elm trained birds

## 2 MATERIALS AND METHODS

### 2.1 Saplings and their treatments

In our experiment, we aimed to test the response of naïve and trained great tits chicks (*Parus major*) to two plant species with qualitatively and quantitatively different herbivore‐induced volatile compounds, which share only few typical HIPVs compounds. Aiming for wide range of potentially evolutionary novel as well as familiar HIPVs to be offered to birds, we selected two plants which are evolutionary distant and very different in terms of HIPVs bouquets. One of them was Scotch elm, *Ulmus glabra* Huds. (*Ulmaceae*), and the second one Cattley guava *Psidium cattleyanum* Sabine *(Myrtaceae*). Scotch elm can be considered as an evolutionarily familiar tree species to great tits. It naturally occurs in the great tit distributional range and hence has been in contact with the birds for centuries. Cattley guava was used as an evolutionary novel plant species; it has not had any evolutionary contact with our experimental bird species. *Psidium* is native to tropical America and the Caribbean and has spread as a crop in subtropical and tropical Asia, tropical Africa, and Oceania. Since the middle of the 20th century, several *Psidium* cultivars have been commercially grown in southwestern Europe and Greece. Saplings (1.5 m tall) of Cattley guava and Scotch elm were planted in 20l pots using a standard agricultural soil 2 months prior to the beginning of the experiment. Of each of the two plant species, 20 individuals were planted; 10 of them were assigned to herbivore‐induced (experimental) treatment and 10 of them to noninduced (control) treatment. To ensure that control saplings did not receive chemical signals from induced saplings, control and induced saplings were placed in separate greenhouses prior to the start of the experiment. All saplings were watered on a weekly basis.

To prepare the induced version of a sapling (i.e., those which will produce herbivore‐induced volatile compounds), we slightly scratched 10 leaves per sapling with a razor and applied saliva of *Locusta migratoria* on them. A combination of mechanical wounding and insect saliva is a commonly employed technique to simulate a controlled level of herbivory (Li et al., 2019). *Locusta migratoria* was selected because of their generalism, which was needed for standardization of our experiment, and global distribution overlapping with distribution of both plant species used in the experiment. *L. migratoria* represents one of few herbivore species that would naturally feed both on elms and guavas. Another reason for its selection was its practicality; they provide large amounts of saliva and can be commercially purchased in large quantities. Although *L. migratoria* is not a typical prey of great tits in Czech Republic, they represent a generalist herbivore which is preyed upon by them, especially in shrubby and grassland landscapes of Europe (Mullié, 2009; Vesey‐FitzGerald, 1955). While we admit that the selected study system does not perfectly reproduce interactions with high relevance in the wild, it represents an ecologically relevant option, which leads to production of plant‐specific but not herbivore‐specific HIPVs attractive to generalist bird species.

After 30 min, damaged leaves were removed. Based on our preliminary tests with this specific study system, this ensured that the plants started to produce HIPVs, but that physical damage was not visible to the birds (see also Mrazova & Sam, 2018). For training as well as experiments, new saplings were prepared this way every four hours to provide the birds with fresh odors. In total, we used 20 individuals of each plant species.

### 2.2 Birds and their training

We used 30 naïve adult great tits as our model insectivorous bird species. Chicks were collected from nest boxes when they were 10–13 days old, between 22 and 24 of May 2018. All chicks from a given nest (4–6 chicks per nest) were kept together in one cage until fledging. Cages were 0.7 × 0.4 × 0.5 m in size with three perches. The birds were kept under natural winter daylight conditions (light from 8:15 to 17:00) controlled by fluorescent light tubes and automatic window shades. After fledging, birds were moved to cages in groups of two to three birds, irrespective of their sex. Up until then, chicks were individually hand‐fed every half‐hour on a diet of mealworms and homemade baked food. The bird food was made by mixing of complete bird food for hand‐raising (Nutribird a21, Versele‐Laga, Belgium), commercial chicken food (Country's Best Show 1 crumble, Versele‐Laga, Belgium), eggs, wheat flower, sugar, and sunflower margarine together and baking at ~180°C for ~40 min. Feeding took place between 6:45 a.m.–8:15 p.m. every day. Hand feeding was continued after fledging, but in intervals of 1 hr, and later 4 hr. This stimulated birds to feed by themselves. At about 35 days after hatching, birds were independent and were relocated to individual cages. Here on after, the homemade baked food, mixed seeds, commercial dried insects (Nobby Orlux Insect Patee Premium, Versele‐Laga, Belgium), and water were provided ad libitum. Mealworms were offered only as supplementary food, so the usually neophobic birds do not refuse them during training. During the whole period of captivity, chicks from different nests could hear and see each other, since they were raised in the same room.

None of the birds used in the experiment had previous direct experience with foliage or HIPVs except that they could have been indirectly exposed to some plant cues associated with caterpillars and other herbivorous insects fed to them by their parents in the first 10–13 days after hatching. The bird chicks came from nest boxes placed in mixed oak‐spruce forest, with no Scotch elm trees present in the surroundings. As HIPVs are plant‐specific, we can consider that the birds used in our experiment are naïve to HIPVs emitted by both sapling species used in the experiment. While Cattley guava represented a completely novel signal to the birds, Scotch elm represented a signal to which the birds could be evolutionarily adapted.

Birds were randomly assigned into three training groups: (1) to associate food with the HIPVs of the Cattley guava, (2) to associate food with the HIPVs of the Scotch elm, and (3) not trained to associate food with any foliage. We performed all training, and experiments, in two Y‐shaped aviaries built with mesh screens (Figure S1). Two 2 m tall dead Pedunculate oaks in a large pot were placed in the corners of the aviary and served as a perch. The birds that were conditioned to find food on induced Cattley guava were offered one induced Cattley guava with 5 mealworms pinned to the leaves and one noninduced Cattley guava without any mealworms. The position of the induced and noninduced Cattley guava (left or right corner) in the aviary was selected randomly. For 1.5 hr every day, birds were allowed to habituate to the aviary and search for food, thus learning to associate the volatile compounds of guava with the reward. The birds were released into the aviary in pairs in the first 3 trials. This made the learning process faster as they could learn from each other. The above process was repeated with elm saplings to condition birds to elm HIPV’s. To condition the control birds to the cage, and initiate their interest in searching for food, we pinned 5 mealworms directly on one of the dead Pedunculate oaks. We conducted one training trial per bird daily. After each training trial, we checked how many meal worms were eaten. We considered the birds to be conditioned to the volatile compounds of the specific plant after they repeatedly ate at least 4 out of the 5 mealworms from the induced sapling.

It took 15 trials to condition all the birds to the induced saplings successfully. Some (40%) birds did not pay too much attention to the plants during the first five trials but investigated mostly the aviary netting and surroundings. After the first five training trials, most of the birds started to eat some mealworms, while some 20% of the birds took at least 10 trials until they started eating mealworms. After the 15th trial, all the birds successfully ate mealworms from the induced saplings. This contrasted with previously described similar training, during which birds were able to find the larvae on sapling after fifth acclimatization (Amo, Jansen, et al., 2013).

### 2.3 Experimental design and procedure

To examine whether great tits were able to learn the specific volatiles of the plant species and associate it with food, we performed a two‐choice experiment. The same aviaries as described above were used. We offered all possible pairwise combinations of plant treatments, to birds with all types of training, except control plants‐control training. This comprised five plant combinations and three bird training treatments (Table 1). All birds participated in all five experiments, that is, we conducted 145 trials in total.

**TABLE 1:** Overview of the experimental design using three groups of birds, 29 in total, and five combinations of saplings, totaling 145 trials at 20 min each

Birds trained to	Experiment 1 Induced elm Control elm	Experiment 2 Induced guava Induced elm	Experiment 3 Induced elm Control guava	Experiment 4 Induced guava Control elm	Experiment 5 Induced guava Control guava
Elm	10	10	10	10	10
Guava	9	9	9	9	9
Naïve	10	10	10	10	10

All experimental trials were performed for 5 days (20–28 Aug 2018), between 09:00 and 17:00, under sunny and warm conditions to avoid variation in the emission of volatiles due to differences in ambient conditions such as temperature (Vallat et al., 2005). On each experimental day, a new pair of saplings was placed in the aviary prior to the first trial and then replaced by a new pair of saplings after every 4 hr. In contrast to training trials, there were no mealworms pinned to any of the saplings during the experiment. Apart from that, the saplings were prepared the same way as for the training. We conducted two 20 min long trials in two separate aviaries simultaneously. Prior to each experimental trial, the birds were starved in their housing cages for 90 min. This ensured that the birds were motivated to search for larvae on the saplings in the experimental aviary. After each trial, the bird was captured with a net and returned to its cage. We recorded the behavior of birds in the aviary during the trials using a video camera (Panasonic Full HD V180EP‐K). One observer, unaware of the treatment, analyzed the video tapes and recorded the time spent on each of the saplings (i.e., in the foliage or on the trunk inspecting the foliage) and the time spent paying attention to the sapling while being within 50cm proximity of the plant (i.e., on the netting of the cage or on the pot). The first 3–5 min of the video recording was discarded to account for the acclimatization of the bird to its environment. Within the first minutes, the bird usually flew around a bit or landed on the wall of the aviary. After a while, the bird perched somewhere, ruffled its feather, and started to look around more slowly. This behavioral change was easy to spot by the observer of the recordings. Experimental trials started every 30 min.

### 2.4 Chemical analyses

We performed gas chromatography to quantify the sampled VOCs (volatile organic compounds). We sampled the volatile compounds of the two plant species by passive trapping with polydimethylsiloxane (PDMS) tube cuttings following a protocol described by Kallenbach et al. (2015) and Klimm et al. (2020) with slight modifications. Using the PDMS tubes, we sampled volatile compounds from ca. half of the saplings. First, we attached the tubes by copper wire to the experimental sapling. We then tightly enclosed as many leaves as possible and the PDMS tube into a polyamide oven bag. We collected the VOCs from the saplings for 24 hr. We removed the tubes, placed them into sterile glass vials, and took them to laboratory for further analyses. The PDMS cuttings were analyzed by a thermal desorption‐gas chromatograph‐mass spectrometer (TD‐GC‐MS) consisting of a thermodesorption unit (MARKES, Unity 2, Llantrisant, United Kingdom) equipped with an autosampler (MARKES, Ultra 50/50). PDMS cuttings were transferred to empty stainless steel tubes (MARKES) and desorbed with helium as a carrier gas and a flow path temperature of 160°C using the following conditions: dry purge 5 min at 20 ml/min, prepurge 1 min at 10 ml/min, desorption 8 min at 200°C with 60 ml/min, trap temperature 0°C, pretrap fire purge 1 min at 60 ml/min, split flow 20 ml/min, trap heated to 230°C and hold for 4 min. The VOCs were separated on a gas chromatograph (Bruker, GC‐456, Bremen, Germany) connected to a triple‐quad mass spectrometer (Bruker, SCION) equipped with DB‐WAX column: (30 m × 0.25 mm inner diameter × 0.25 µm film thickness, Restek). The temperature program was the following: 60°C (hold 1 min), 30°C/min to 150°C, 10°C/min to 200°C and 30°C/min to 230°C (hold 1 min). Helium was used as a carrier gas at a constant flow rate of 1 ml/mi. Mass spectrometer conditions were set at a 40°C manifold, 240°C transfer line, and 220°C for the ion source. The scan range was 33–500 m/z for a full scan and scan time was 250 ms. We selected the most prominent peaks in the chromatograms (signal to noise ratio >10). Peaks that were also present in air blanks were regarded as systemic contamination and were excluded from further analysis. VOCs were tentatively identified by comparison to the NIST database and comparison to retention indices from the literature. The annotation of the most significant features was done by spectral library search (NIST) and MS spectra and Kovats Index comparison with standard compounds. The peak areas of these compounds were calculated using the Bruker Workstation software (v8.0.1).

## 3 STATISTICAL ANALYSIS

We used the proportion of time spent on each of the saplings for the analysis. We used the glmmTMB package (Brooks et al., 2017) within R 3.6.1 (R Core Team, 2019) to build a generalized linear mixed model with a beta error structure and a logit link. Models that follow beta distribution only allow proportion values between 0 and 1. Therefore, 0 and 1 values in our study were converted into 0.0001 and 0.9999, respectively. We used the mixed model to determine the effect of experiment (sapling combination), bird training, and their interaction on the proportion of time spent by an individual bird on sapling. We used the ANOVA function from the “car” package to calculate *p*‐values for each variable using a Wald test (Fox & Weisberg, 2018). We then made pairwise comparisons between the five experiments within each of the training types using the *emmeans* functions in “emmeans” package that adjusts *p*‐values (*p*_adj_) following the Tukey method (Lenth, 2007).

The centroided GC‐MS data in NetCDF format were further processed using XCMS online (version 2.7.2). The peaks in each sample were detected with the cent Wave algorithm (peak width 2–20 s; signal to noise threshold 10). Peak grouping across samples was restricted to peaks present in at least 50% of the samples in at least one treatment group (minfrac = 0.5). Retention time correction was accomplished with the symmetric method and nonlinear loess‐smoothing and iterated three times with decreasing bandwidth parameter for the grouping from 10 to 0.2 s. The extracted ion species were grouped according to their parent molecule into pseudospectra with the Bioconductor package camera (Kuhl et al., 2012). This resulted in a final feature table containing mass‐to‐charge ratio (m/z), retention time, peak area, and the pseudospectra group for each detected ion species. Groups containing only a single ion species were artifacts and removed from the final feature table. The feature table was uploaded to MetaboAnalyst for further statistical analyses. We performed quantile normalization and Log transformation. Pareto Scaling was chosen for principal component analysis.

## 4 RESULTS

### 4.1 Results of behavioral tests

The proportion of time spent by individual birds on a sapling was significantly dependent on sapling type, bird training, and their interaction (X^2^ = 31.62, *p* = .001). This result suggests that the statistical difference between experiments is dependent on the type of training given to the individual birds. Naïve birds (control) spent around 50% of their time on each of the two saplings in four out of the five experiments (Figure 2; Table S1).

Elm trained birds spent significantly longer time on induced elm saplings than noninduced elm saplings (Figure 2). The time spent on each sapling varied significantly across experiments (Table 1). In experiments where herbivore‐induced elm saplings were offered to birds together with noninduced elm control (Exp 1) or noninduced guava (Exp 3), birds spent, respectively, 82% (69%–91%) and 75% (58%–87%) of their time on the induced saplings. In experiment 2, 4, and 5, birds spent equal amounts of time on each treatment (Figure 2). When induced elm saplings were offered to birds together with induced guava saplings in experiment 2, birds only spent c. 56% (25% to 64%) on induced elm. Experiments 4 and 5 did not contain induced elm saplings but contained induced guava sapling and the birds did not show any specific preference (Figure 2; Table S2).

Guava trained birds spent a significantly greater amount of time on herbivore‐induced saplings than noninduced saplings (Figure 2). The time spent on each sapling varied significantly across experiments (Table S3). In experiments 4 and 5, where herbivore‐induced saplings were presented with noninduced saplings, birds spent c. 82% (67%–91%) and 76% (58%–88%) of their time on those saplings. In experiment 2, where herbivore‐induced guava saplings were presented with herbivore‐induced elm saplings, birds spent c. 71% (52%–85%) of their time on induced guava saplings. In experiments 1 and 3 where herbivore‐induced elm saplings were presented, birds did not show any specific preference (Figure 2).

### 4.2 Induced volatiles of experimental plants

We detected significantly more chemical compounds in induced than in noninduced guavas (*p* = .009, Figure S2). Compounds found in significantly higher amounts were β‐Ocimene, Heptadiene, Cyclohexane, α‐Pinene, Copaene, Caryophyllene, and Tetraline. The induced elm also produced more compounds than the noninduced elm; the difference however was not statistically significant (Figure S3). The induced elm had significantly higher abundances of β‐Ocimene, α‐Farnesene, Tetraline, 1‐Butanol, 3‐methyl‐, acetate, and 2‐Butanone,4‐(2,6,6‐trimethyl‐1‐cyclehexene‐1‐yl) compared to the noninduced elm. Considering the most significant volatile compounds of each sample, difference between the two plant species was bigger than difference within the species (Figure S4). Significant increase of β‐Ocimene and Tetraline was feature shared between the herbivore‐induced elms and guavas.

## 5 DISCUSSION

Our results brought further support for previous suggestions that attraction of great tits to herbivore‐induced defense cues is not an innate trait. Naïve birds with no experience with foliage were not attracted to induced, thus potentially insect rich, saplings. This confirms the previous findings of Amo et al. (2016). In their experiment, naïve birds neither preferred the caterpillar‐infested sapling during the first visit, nor visited that sapling more frequently than the uninfested sapling (control). In contrast, in another study, when the great tits gained some experience with foraging for caterpillars, they exhibited a preference for caterpillar‐infested saplings and visited them more often than the uninfested saplings (Amo, Jansen, et al., 2013). In line with these results, we were able to teach the birds the association between the herbivore‐induced volatile compounds of saplings and a food reward.

Contrary to our expectation, each group of trained bird preferred the herbivore‐induced sapling of the plant species they were trained to, over any other saplings offered to them. The birds did not seem to be able to generalize the learned bouquets of volatile compounds to other plant species. The learning process was flexible, as the great tits were able to associate the HIPVs of tropical guava with food to a similar extent, if not better, as the HIPVs of elm. This might imply that the birds are likely to learn the whole bouquets of the volatile compounds and associate them with food, rather than learning individual volatile compounds. An alternative explanation might be that our design failed to reveal the potential existence of a generalization based on other compounds than the ones shared between the two plant species tested by us. In this respect, the birds can undertake some form of generalized learning; familiar HIPVs may help with the learning of similar HIPVs from closely related plant species.

We detected a significantly greater number of chemical compounds in induced compared to noninduced guavas. The number of produced compounds was higher in induced than in noninduced elms, but the difference was not significant. Compounds found in significantly higher concentration in induced plants were β‐Ocimene, Heptadiene, Cyclohexane, α‐Pinene, Copaene, Caryophyllene, Tetraline, α‐Farnesene, and Tetraline, but only β‐Ocimene and Tetraline was significantly increased after induction in both plant species. Some of these compounds have been thought to play a role in attracting birds to herbivore‐damaged plants, especially β‐Ocimene and α‐Pinene (Mrazova et al., 2019). Our experiment showed that birds exposed to two plants with very different bouquets of HIPVs can distinguish their treatments (induced/noninduced) from each other better than birds exposed to more similar bouquets. In our experiment, we selected two very different plant species; one tropical plant with a strong odor and one temperate plant with a rather weak odor. Our birds were not able to generalize between their odors, despite they shared increased amounts of β‐Ocimene and Tetraline, two out of several compounds suspected to be used by generalist insectivorous predators. The question remains as to whether the birds would be capable of generalizing between several temperate plant species which might have more similar odors or at least different shared compounds.

In contrast to the results in a recent study by Amo et al. (2016), our birds took a long time to successfully search for the mealworms offered to them on induced saplings. Amo et al. (2016) found that birds quickly learnt to use HIPVs as a foraging cue, associating the presence of caterpillars with the HIPVs of infested trees within 5 hr (Amo et al., 2016). Similarly, in our previous study, naïve birds were able to locate more than 80% of caterpillars after 5 hr of habituation in aviary conditions with noninduced trees (Mrazova et al., unpublished). In the current study, bird needed on average at least 20 hr to be able to associate the food‐rich sapling with the volatile compounds. The difference between the previous and current studies was the type of the sapling offered during the training phase; Amo et al. (2016) offered two infested saplings, and Mrazova et al. (unpublished) offered two uninfested saplings, compared to the current study where one infested and one uninfested sapling were offered, and the training was considered to be successful only after the birds ate from induced saplings.

Learning of olfactory signals can occur in birds actively after hatching if the birds learn to associate the odors with a reward, or even passively before hatching. Prenatal chemosensory learning has been shown in domestic chickens (Sneddon et al., 1998). Upon no exposure to strawberries before hatching, chickens were highly aversive to strawberries after hatching. However, following exposure to strawberries before hatching, chicks expressed a greater preference for, or weaker aversion to, the strawberry stimulus (Sneddon et al., 1998). This shows that chicks can learn and prefer a particular smell. Within just a few hours after hatching, chicks can learn the olfactory cues of their nest, using them later for nest recognition (Caspers et al., 2013).

Prey availability for birds fluctuates throughout the year due to phenological changes of the plants and insect species they rely on. Adaptive plasticity may therefore be an advantage for foraging birds in response to changes in distribution and abundance of prey species (Murakami, 1998). In this study, we have shown that great tits are able to learn evolutionarily novel odors and associated different odors with specific food resources. This may maximize foraging success in birds as they utilize novel plant species in their environment. Additionally, we have shown that generalized learning of bouquets of HIPVs occurs. It is important to note that our study was conducted on a single bird species; the adaptive value of learning is expected to vary among bird species depending on their diet breadth at both the herbivore and plant level (Mrazova et al., 2019; Vet et al., 1995). Additionally, the use of olfaction in foraging can be innate in some bird species (Amo, Rodríguez‐Gironés, et al., 2013; Bonadonna et al., 2006). The use of olfaction can also depend on the type of cue. Innate attraction to a single compound (e.g., atmospheric dimethyl sulfide for marine birds) can be arguably easily achieved during evolution, whereas the HIPVs are so variable that it might be difficult to have their recognition innate in generalist predators.

In our study, we also used two phylogenetically distant tree species which differed significantly in their diversity of herbivore‐induced volatile compounds. It is possible that some form of generalized learning by birds takes place in order to recognize volatile compounds of phylogenetically related tree species, but our design was not able detect it. Assuming however that associations of whole complex odors and prey need to be learned, temperate birds may have the situation easier due to smaller variety of plant species in their home range. Same learning would represent a challenge for tropical birds whose home ranges can consist of many hundred phylogenetically diverse tree species (Anderson‐Teixeira et al., 2015). Tropical insectivorous understory birds usually have home ranges up to 30 ha large. A hectare of tropical forest typically contains more than 250 tree species (Anderson‐Teixeira et al., 2015), and many of them carrying potential prey items. It might be difficult for birds to learn the HIPVs from so many herbivore‐induced tree species, especially if their HIPVs differ significantly, so perhaps they learn only a subset of local plants or groups of plants which are chemically similar and therefore also phylogenetically related. Further research should focus on responses of birds to more phylogenetically variable plant species, which overlap and differ in many more HIPVs than our two plant species. Our research was not only limited to one pair of plant species but also to a single bird species, which should be also improved in further research. It remains unknown how the learning of volatile compounds works in other bird species with different searching strategies, especially those from rich tropical areas, and how is the ability to learn volatile compounds combined with ability to learn the visual signals, which also different between bird species.

## CONFLICT OF INTEREST

Authors declare no conflict of interest.

## AUTHOR CONTRIBUTION

**Katerina Sam:** Conceptualization (lead); Data curation (lead); Formal analysis (supporting); Funding acquisition (lead); Methodology (lead); Project administration (lead); Supervision (lead); Visualization (supporting); Writing‐original draft (lead); Writing‐review & editing (equal). **Eliska Kovarova:** Data curation (equal); Investigation (equal); Methodology (equal); Writing‐review & editing (equal). **Inga Freiberga:** Data curation (equal); Investigation (equal); Methodology (equal); Project administration (equal); Writing‐review & editing (equal). **Henriette Uthe:** Formal analysis (supporting); Investigation (supporting); Validation (supporting); Visualization (supporting); Writing‐review & editing (supporting). **Alexander Weinhold:** Conceptualization (supporting); Formal analysis (supporting); Investigation (supporting); Visualization (supporting); Writing‐review & editing (supporting). **Leonardo Re Jorge:** Data curation (supporting); Formal analysis (supporting); Validation (supporting); Writing‐review & editing (supporting). **Rachakonda Sreekar:** Formal analysis (lead); Investigation (supporting); Validation (equal); Visualization (lead); Writing‐review & editing (supporting).

## Supporting information

Supplementary Material

Supplementary Material

## Data Availability

Raw data and R scripts are available for download from Dryad Digital Repository on link: https://datadryad.org/stash/share/Gja7gg16XSCvHE7uqka7ZS4bYtgyN0WKvUjkE7wxVG8 R‐code is also attached as [Link], [Link].
