# Supplementary material for: Great tits (Parus major) flexibly learn that herbivore‐induced plant volatiles indicate prey location: An experimental evidence with two tree species
Source: Ecol Evol. 2021 Jul 21;11(16):10917–25. doi: 10.1002/ece3.7869 (PMC8366880; doi:10.1002/ece3.7869)

Supplementary material: Great tits (*Parus major*) flexibly learn that herbivore-induced plant volatiles indicate prey location – experimental evidence with two tree species

Figure S1. Experimental aviary - The aviary always contained three perches, one in the middle of the rear side of the aviary and two dead, ca. 1.7m tall, Pedunculate oaks (*Quercus robur*) which served as perches in the corners of the rear side of the aviary. The experimental part of the aviary had dimension 4 x 2.5 x 1.5 x 2.5 (rear, side, front, rear length of the wall) and was connected to an entry space (1.5 x 1.5 m).


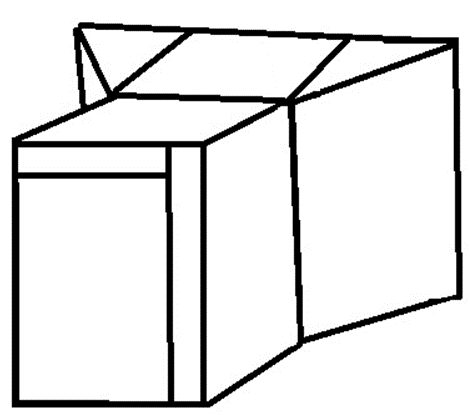


Figure S2. PCA of the VOCs produced by the induced guava (Treat) samples collected 30 min and 3.5 hours after induction and control guava (CTRL) samples collected 3 hours apart. There was no significant difference in VOCs in different times, so the different times of the collection were summarized.


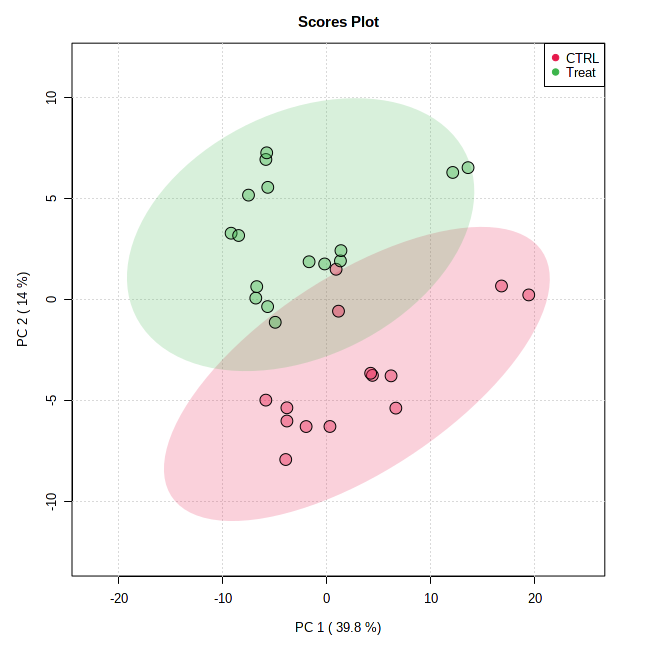


Figure S3. PCA of the VOCs produced by the induced elm (Elm_Treat) samples collected 30 min and 3.5 hours after induction and control elm (Elm_CTRL) samples collected 3 hours apart. There was no significant difference in VOCs in different times, so the different times of the collection were summarized.


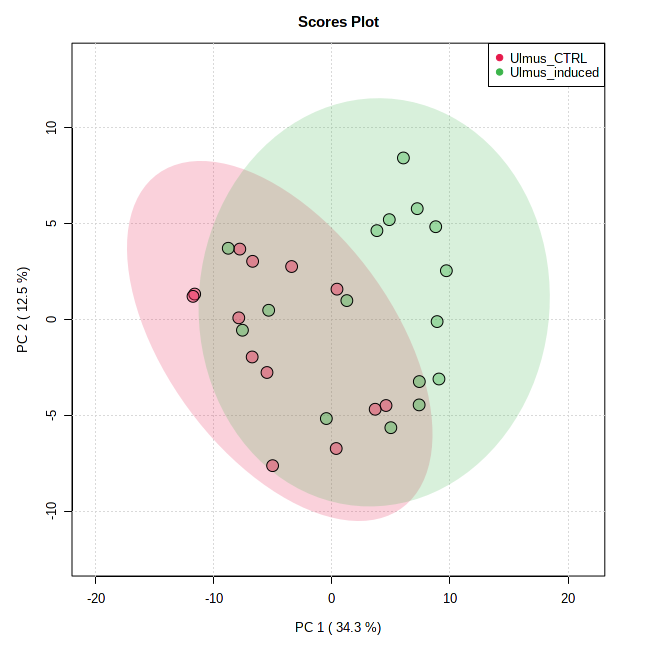


Figure S4. PCA of the VOCs produced by the induced elm samples collected 30 min after induction (JIA) and 3.5 hours after induction (JIB) and control elm samples collected 3 hours apart (JA and JB) and by induced guava samples collected 30 min after induction (PIA) and 3.5 hours after induction (PIB) and control guava samples collected 3 hours apart (PA and PB).


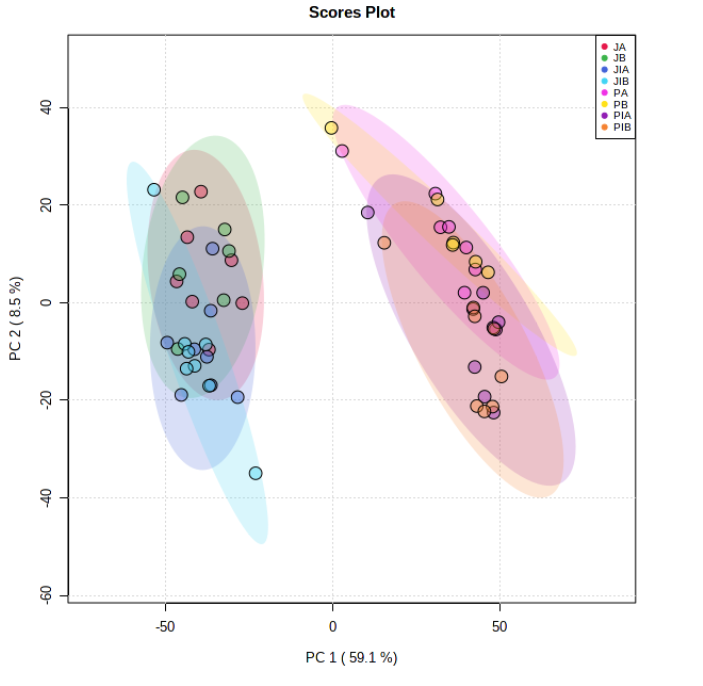

Supplement: Supplementary file 1 — Supplementary Material [file ECE3-11-10917-s001.docx]
