# Supplementary material for: Great tits (Parus major) flexibly learn that herbivore‐induced plant volatiles indicate prey location: An experimental evidence with two tree species
Source: Ecol Evol. 2021 Jul 21;11(16):10917–25. doi: 10.1002/ece3.7869 (PMC8366880; doi:10.1002/ece3.7869)
Supplement: Supplementary file 2 — Supplementary Material [file ECE3-11-10917-s002.docx]

Supplementary material: Great tits (*Parus major*) flexibly learn that herbivore-induced plant volatiles indicate prey location – experimental evidence with two tree species

Script 1. Script for R program

Raw data available in DRYAD repository under accession number:

dat <- read.csv("~/Naive_Birds/CompleteData.csv")

library(lme4)

library(sjPlot)

library(sjmisc)

library(ggplot2)

library(snakecase)

library(ggeffects)

library(emmeans)

library(glmmTMB)

library(DHARMa)

summary(dat)

library(car)

dat$prop<-dat$TimeClose/dat$TotalTimeCloseTree

dat$prop[is.na(dat$prop)] <- 0

dat$prop[dat$prop == 0] <- 0.0000001 # No zeros allowed

dat$prop[dat$prop == 1] <- 0.9999999

summary(dat)

dat$Trained.to <- factor(dat$Trained.to,levels = c("Control", 'Psidium', 'Ulmus'))

m1<-glmmTMB(prop~Experiment*Trained.to+(1|Bird),family=beta_family(link = 'logit'),data=dat)

summary(m1)

Anova(m1)

cols<-c('Control'='black', 'Psidium' = 'grey40', 'Ulmus' = 'grey75')

library(emmeans)

emmeans(m1,pairwise ~ Experiment | Trained.to)

ggpredict(m1)

dat$Trained.to <- factor(dat$Trained.to,levels = c("Psidium", 'Control', 'Ulmus'))

m1<-glmer(cbind(TimeClose,TotalTimeCloseTree-TimeClose)~Experiment*Trained.to+(1|Bird),family='binomial',data=dat,control=glmerControl(optimizer="bobyqa",optCtrl=list(maxfun=2e5)))

ggpredict(m1)

dat$Trained.to <- factor(dat$Trained.to,levels = c( 'Ulmus',"Psidium", 'Control'))

m1<-glmer(cbind(TimeClose,TotalTimeCloseTree-TimeClose)~Experiment*Trained.to+(1|Bird),family='binomial',data=dat,control=glmerControl(optimizer="bobyqa",optCtrl=list(maxfun=2e5)))

ggpredict(m1)

##Figure

datr <- read.csv("~Documents/Manuscript/Naive_Birds//Katka naive birds_v2.csv")

library(ggplot2)

theme_katka2 <- function (base_size = 12, base_family = "") {

theme_gray(base_size = base_size, base_family = base_family) %+replace%

theme(

panel.grid.minor = element_blank(),

panel.grid.major = element_line(colour = "grey80",size=0.2),

axis.title.x = element_text(colour = "black", size=rel(1.5)),

panel.background = element_rect(fill="white"),

axis.title.y = element_text(colour = "black", size=rel(1.5),angle=90,vjust=0.3),

axis.text.x= element_text(colour = "black", size=rel(1.5)),

axis.text.y= element_text(colour = "black", size=rel(1.5))

)

}

theme_set(theme_katka2())

#fill <- c("#56B4E9", "#5F9EA0","#b2d183","#E1B378")

#fill = c("#56B4E9", "#b2d183", "#E7B800", "#CC79A7")

#fill = c("orange", "cadetblue2", "darkorange3", "deepskyblue3")

fill = c("deepskyblue3", "darkorange3", "cadetblue2","orange")

ggplot(data = datr) +

geom_bar(data = datr, aes(x = Experiment, y = Estimate, fill=Treatment), stat="identity") + scale_fill_manual(values=fill) +

geom_errorbar(data = datr, aes(x = Experiment, y = Estimate, ymin = Low, ymax = High, width = 0.001)) +

xlab('') + ylab('') + facet_wrap(~Training) + geom_hline(yintercept=0.5,color='grey40', linetype='dashed', size =1) +

theme(axis.title.x=element_text(vjust=-0.2),axis.title.y=element_text(hjust=0.4), strip.text = element_text(size=20))

### Induced vs Non-induced

dat <- read.csv("~/Downloads/CompleteData.csv")

m2<-glmmadmb(prop~Exp*Tr.to+(1|Bird),family='beta',data=dat)

summary(m2)

plot_model(m2, type = "pred", terms = c('Tr.to','Exp')) + xlab('') + ylab('') + ggtitle('')

ggpredict(m2)

dat$Tr.to <- factor(dat$Tr.to,levels = c("Induced", 'Control'))

m2<-glmer(cbind(TimeClose,TotalTimeCloseTree-TimeClose)~Exp*Tr.to+(1|Bird),family='binomial',data=dat,control=glmerControl(optimizer="bobyqa",optCtrl=list(maxfun=2e5)))

ggpredict(m2)

fill2 = c( "grey70", "grey30")

datr2 <- read.csv("~/Documents/Post-PhD stuff/Birds Naive Katka/naive_estimates_not_originial.csv")

ggplot(data = datr2) +

geom_bar(data = datr2, aes(x = Experiment, y = Estimate, fill=Treatment),colour="black", stat="identity") + scale_fill_manual(values=fill) +

geom_errorbar(data = datr2, aes(x = Experiment, y = Estimate, ymin = Low2, ymax = High2, width = 0.001)) +

xlab('') + ylab('') + facet_wrap(~Training) + geom_hline(yintercept=0.5,color='grey40', linetype='dashed', size =1) +

theme(axis.title.x=element_text(vjust=-0.2),axis.title.y=element_text(hjust=0.4), strip.text = element_text(size=20))
